# Inflammation and Thrombophilia Markers in Supra-Aortic Takayasu Arteritis-Associated Stroke: A Digital Subtraction Angiography-Based Case Control Study

**DOI:** 10.3390/jcm15062308

**Published:** 2026-03-18

**Authors:** Ebru Marzioglu Ozdemir, Gokhan Ozdemir

**Affiliations:** 1 Department of Medical Genetic, Selcuk University, Konya 42130, Turkey; ebru.ozdemir@selcuk.edu.tr; 2 Department of Neurology, Center of Stroke, Selcuk University Medical Faculty, Konya 42130, Turkey

**Keywords:** Takayasu arteritis, stroke, inflammation, thrombophilia, lipoproteins, homocysteine, digital subtraction angiography

## Abstract

**Background/Objectives**: Takayasu arteritis is an important non-atherosclerotic cause of ischemic stroke in young adults. However, the relative contribution of systemic inflammation, inherited thrombophilia, and supra-aortic hemodynamic impairment to cerebrovascular events in these patients remains insufficiently defined. This study aimed to evaluate the relative impact of systemic inflammatory activity, hereditary and acquired thrombophilia markers, and supra-aortic vascular involvement on cerebrovascular ischemic events in patients with digital subtraction angiography (DSA) confirmed supra-aortic Takayasu arteritis. **Methods**: A retrospective cross-sectional analysis was conducted in consecutively evaluated patients with non-atherosclerotic inflammatory stenosis or occlusion of the carotid, subclavian, or vertebral arteries confirmed by digital subtraction angiography. Age- and sex-matched hospital-based individuals without autoimmune, thrombotic, or cerebrovascular diseases served as controls. Laboratory assessments including erythrocyte sedimentation rate, lipoprotein(a), homocysteine, antinuclear antibody, rheumatoid factor, antiphospholipid antibodies, and a hereditary thrombophilia panel were obtained 4–6 weeks after clinical presentation during a stable clinical phase. **Results**: Among 46 patients with Takayasu arteritis, 21 patients presented with ischemic stroke. The stroke-positive subgroup demonstrated higher inflammatory activity and a slightly greater prevalence of supra-aortic occlusive lesions, particularly involving the common carotid, internal carotid, and subclavian arteries. Although lipoprotein(a) levels showed statistical differences between groups, mean values remained within reference ranges and were not clinically elevated. The distribution of hereditary thrombophilia variants and the prevalence of elevated homocysteine levels did not differ significantly between groups. Clinical outcomes were favorable overall, with no mortality and functional independence achieved in the majority of stroke-positive patients. **Conclusions**: These findings suggest that systemic inflammation and supra-aortic hemodynamic impairment may play a more prominent role than inherited thrombophilia in the development of cerebrovascular ischemic events in patients with Takayasu arteritis. Selective rather than routine thrombophilia testing may therefore be appropriate in selected clinical contexts, while careful control of inflammatory activity and continuous vascular monitoring remain essential components of management.

## 1. Introduction

Takayasu arteritis is a chronic, idiopathic large-vessel vasculitis involving the aorta and its major branches, characterized by inflammatory stenosis, occlusion, or aneurysmal dilatation [1]. The disease is more common in Asian and Middle Eastern ethnicities and primarily affects young women [2,3,4]. Vascular involvement frequently includes the supra-aortic branches, resulting in compromised cerebral inflow and increased susceptibility to cerebrovascular ischemia [5].

Cerebrovascular ischemic events, such as ischemic stroke and transient ischemic attack, have been seen in a clinically significant subset of patients with Takayasu arteritis in population-based cohorts and large multicenter studies, especially those with supra-aortic involvement (Numano type I, defined as disease limited to the branches of the aortic arch) [6,7]. In this setting, cerebral ischemia is thought to arise predominantly from two interrelated mechanisms: (i) inflammation-driven luminal narrowing of the carotid, subclavian, or vertebral arteries, resulting in impaired cerebral inflow and reduced cerebrovascular reserve, and (ii) superimposed thrombotic or embolic events developing on an inflamed and structurally damaged endothelium under conditions of disturbed flow [8,9]. While inflammatory vascular remodeling is widely regarded as the primary driver of arterial injury in Takayasu arteritis, individual susceptibility to ischemic events may be further modulated by additional prothrombotic factors [10,11]. In the general young adult population, both inherited and acquired thrombophilia markers including Factor V Leiden, Prothrombin G20210A, MTHFR polymorphisms, lipoprotein(a), and antiphospholipid antibodies have been variably associated with ischemic stroke in the absence of traditional vascular risk factors [12]. However, insufficient evidence exists to determine whether these thrombophilic characteristics serve as significant ‘second hits’ in the context of inflammation-induced endothelial damage and altered hemodynamics typical of Takayasu arteritis. In such a pro-inflammatory and flow-disturbed vascular environment, it remains unclear whether inherited or acquired prothrombotic traits meaningfully amplify ischemic risk beyond the structural burden imposed by large-vessel stenosis. To date, no study has specifically evaluated the prevalence of hereditary and acquired thrombophilia markers in Turkish patients with Takayasu arteritis, despite the relatively high background frequency of certain prothrombotic polymorphisms in Mediterranean populations.

Accurate evaluation of supra-aortic involvement is essential for both diagnosis and risk stratification in Takayasu arteritis. Digital subtraction angiography provides high-resolution assessment of stenosis severity, occlusion patterns, and collateral circulation, allowing reliable differentiation from atherosclerotic disease or fibromuscular dysplasia [13]. The primary objective of this study was to evaluate whether hereditary and acquired thrombophilia markers contribute independently or additively to cerebrovascular ischemic events in patients with angiographically confirmed supra-aortic Takayasu arteritis. We hypothesized that, in this specific population, systemic inflammation and hemodynamic arterial compromise would demonstrate a stronger association with stroke occurrence than classical hereditary thrombophilia markers. The null hypothesis was that thrombophilia markers would not differ significantly between stroke-positive and stroke-negative supra-aortic Takayasu patients. The primary analytic comparison of this study was between stroke-positive and stroke-negative supra-aortic Takayasu arteritis patients, in order to determine whether thrombophilia markers independently differentiate ischemic presentation within this specific vasculitic population. Comparisons with healthy controls were performed as a secondary contextual analysis to evaluate the background distribution of thrombophilia markers in our regional population.

## 2. Methods

### 2.1. Study Design and Population

This retrospective cross-sectional study was conducted at the Stroke Center of Selcuk University Faculty of Medicine between January 2017 and June 2025 and included all consecutively evaluated patients with supra-aortic Takayasu arteritis. The retrospective design reflects the use of previously recorded clinical, laboratory, and imaging data, while the cross-sectional framework indicates that comparisons between stroke-positive and stroke-negative groups were performed at the time of index presentation, without longitudinal follow-up.

**Figure 1:**
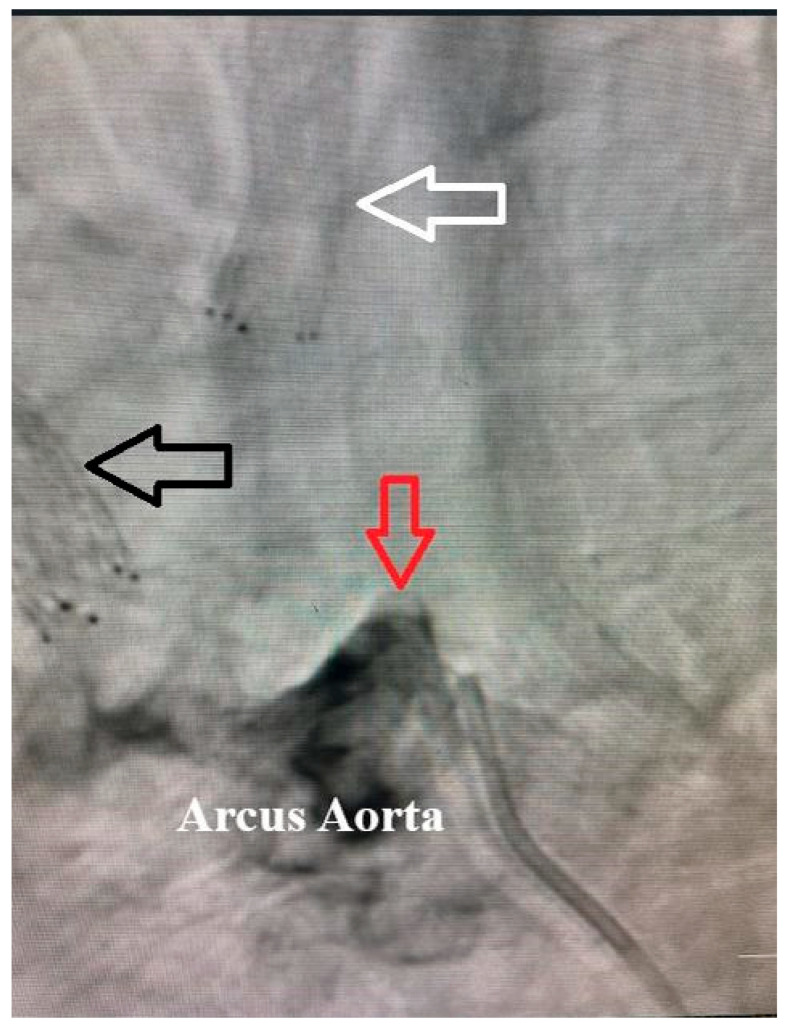
Aortic arch angiography demonstrating supra-aortic arterial involvement in Takayasu arteritis. The aortic arch gives rise to the supra-aortic vessels supplying the brain. The black arrow indicates a previously implanted stent in the brachiocephalic artery. The white arrow denotes a stent placed in the proximal segment of the left common carotid artery due to severe inflammatory stenosis. The red arrow demonstrates complete occlusion of the proximal left subclavian artery following a short arterial stump, consistent with progressive large-vessel involvement.

Patients were included if they fulfilled the 2022 American College of Rheumatology/European Alliance of Associations for Rheumatology (ACR/EULAR) classification criteria for Takayasu arteritis [14] and demonstrated angiographically confirmed supra-aortic arterial involvement. Supra-aortic involvement was defined as non-atherosclerotic inflammatory stenosis or occlusion of one or more branches of the aortic arch, including the common carotid, internal carotid, subclavian, or vertebral arteries (Figure 1).

A systematic, step-by-step method that combined vascular imaging, inflammatory markers, and clinical evaluation was used to diagnose Takayasu arteritis. The reference imaging technique for vascular classification and diagnostic confirmation was digital subtraction angiography (DSA), which was applied consistently. Angiographic features consistent with Takayasu arteritis included long-segment, smooth, concentric stenosis or occlusion, absence of calcified plaques, tapered narrowing, post-stenotic dilatation, and the presence of collateral circulation. These characteristics enabled reliable differentiation from atherosclerotic disease, fibromuscular dysplasia, arterial dissection, and radiation-induced vasculopathy. All included patients were subsequently classified according to the Numano angiographic classification, and all met criteria for Type I disease, reflecting isolated supra-aortic branch involvement.

Laboratory and thrombophilia evaluations were performed uniformly in all participants during a clinically stable period and were not used as inclusion criteria. Assessed parameters included erythrocyte sedimentation rate, lipoprotein(a), plasma homocysteine, antinuclear antibody, rheumatoid factor, antiphospholipid antibodies, and a comprehensive hereditary thrombophilia panel. This standardized diagnostic and inclusion strategy ensured a homogeneous study population with high diagnostic specificity.

### 2.2. Inclusion Criteria

Patients were eligible for inclusion if they met all of the following criteria:

1. Diagnosis of Takayasu arteritis according to the 2022 American College of Rheumatology/European Alliance of Associations for Rheumatology (ACR/EULAR) classification criteria.
2. Angiographically confirmed supra-aortic arterial involvement, defined as non-atherosclerotic inflammatory stenosis or occlusion of one or more supra-aortic vessels (common carotid, internal carotid, subclavian, or vertebral arteries).
3. Confirmation of vascular involvement by digital subtraction angiography, serving as the reference imaging modality.
4. Age ≥ 18 years at the time of diagnosis.
5. Availability of complete clinical, laboratory, angiographic, and thrombophilia data in institutional medical records.
6. For the stroke-positive group, documentation of ischemic stroke confirmed by neuroimaging (computed tomography or magnetic resonance imaging).

### 2.3. Exclusion Criteria

Patients were excluded if any of the following conditions were present:

1. Evidence of atherosclerotic, fibromuscular dysplasia–related, radiation-induced, or dissection-related vascular disease on angiographic evaluation.
2. Diagnosis of secondary vasculitis or other systemic inflammatory or connective tissue diseases.
3. Active infection, malignancy, or pregnancy at the time of evaluation.
4. History of cardioembolic stroke sources, including atrial fibrillation with high embolic risk, mechanical heart valves, or intracardiac thrombus.
5. Ongoing long-term oral anticoagulant therapy at the time of thrombophilia testing.
6. Incomplete clinical records or unavailable laboratory data required for thrombophilia and inflammatory marker analysis.

The study cohort was categorized into three groups:

- Stroke-positive Takayasu arteritis: Individuals exhibiting ischemic stroke.
- Stroke-negative Takayasu arteritis: Patients devoid of cerebrovascular incidents.
- Healthy controls: The control group was age- and sex-matched to minimize potential confounding. The control group consisted of hospital-based individuals without a history of autoimmune, inflammatory, thrombotic, or cerebrovascular disease. Controls were screened by clinical interview and routine laboratory assessment; no vascular imaging was performed. We acknowledge that the relatively high prevalence of hypertension suggests that this group represents a hospital-based reference population rather than a community-based healthy cohort.

### 2.4. Clinical, Neurological Assessment, and Treatment

All patients underwent a standardized clinical evaluation jointly performed by a neurologist and a rheumatologist experienced in cerebrovascular disease and large-vessel vasculitis. Medical history, smoking status, hypertension, diabetes mellitus, dyslipidemia, atrial fibrillation, and other traditional vascular risk factors were all methodically documented. Acute localized neurological impairments were thoroughly documented as part of the neurological examination. In accordance with emergency department protocols, non-contrast computed tomography (CT) of the brain was used as the first-line imaging modality in all patients presenting with suspected acute ischemic stroke, primarily to exclude intracranial hemorrhage and major stroke mimics. Among stroke-positive Takayasu arteritis patients, CT imaging was performed at initial presentation in all cases. Magnetic resonance imaging (MRI), including diffusion-weighted sequences, was subsequently performed during the subacute phase or early follow-up in selected patients to confirm cerebral ischemia, determine infarct location, and assess lesion extent when clinically indicated or available. In patients who did not undergo MRI, the diagnosis of ischemic stroke was established based on compatible clinical findings supported by CT imaging and clinical evolution. The angiographic distribution of Takayasu arteritis was classified according to the Numano angiographic system, and all included patients were categorized as Type I, reflecting isolated involvement of the supra-aortic branches [6]. All patients were managed according to a standardized institutional treatment protocol developed collaboratively by the stroke neurology and rheumatology teams. Therapeutic decisions were guided by clinical presentation (acute versus non-acute), inflammatory activity, and the severity and hemodynamic significance of supra-aortic arterial involvement as assessed by digital subtraction angiography.

1. Acute Stroke Management Protocol: Patients presenting with acute ischemic stroke were evaluated for intravenous thrombolysis according to current international stroke guidelines. In thrombolysis-eligible individuals, standard-dose IV thrombolysis was administered. Regardless of thrombolysis status, all acute-phase patients received dual antiplatelet loading consisting of aspirin 300 mg and clopidogrel 450 mg at admission. High-dose intravenous pulse corticosteroid therapy was initiated in the acute phase (approximately 5 days), followed by transition to oral corticosteroids at 1 mg/kg/day. This regimen aimed to rapidly suppress active vascular inflammation and stabilize hemodynamics before any consideration of revascularization.

2. Immunosuppressive Strategy: Following stabilization, patients were transitioned to steroid-sparing immunosuppressive agents such as methotrexate, azathioprine, or mycophenolate mofetil according to rheumatologic assessment. In cases with more severe clinical presentation or when conventional agents were insufficient, interleukin-6 inhibition (tocilizumab) was considered as an escalation strategy. Initiation and titration of immunosuppressive therapies were guided by inflammatory markers and clinical activity scores.

3. Endovascular Management Protocol: Endovascular treatment (balloon angioplasty or stenting) was not performed during the acute stroke phase. Revascularization was considered only after: completion of the acute inflammatory phase, stabilization with corticosteroids, and improvement in systemic inflammatory markers. Stenting or angioplasty was performed ≥1 month after the index presentation, targeting hemodynamically significant or symptomatic stenotic lesions of the brachiocephalic, carotid, or subclavian arteries.

4. Antiplatelet Management: Patients who underwent endovascular intervention were placed on dual antiplatelet therapy (aspirin 100 mg/day + clopidogrel 75 mg/day) for at least three months, followed by transition to long-term single antiplatelet therapy. Long-term single antiplatelet therapy was recommended for all patients with supra-aortic Takayasu arteritis regardless of stroke status.

### 2.5. Laboratory and Immunological Evaluation

Laboratory and immunological data were retrieved retrospectively from medical records and the institutional laboratory database. All laboratory investigations had been performed as part of routine clinical evaluation in patients with suspected or confirmed Takayasu arteritis and were not prospectively planned for research purposes. Venous blood samples had been collected during routine outpatient or inpatient follow-up after a minimum of 8 h of overnight fasting, and during a clinically stable phase of disease activity, typically 4–6 weeks after the index presentation, in order to minimize the influence of acute inflammatory fluctuations. Standard biochemical and hematologic parameters, including erythrocyte sedimentation rate (ESR) and complete blood count (CBC), were measured using routine automated methods. Rheumatologic markers included antinuclear antibody (ANA) and rheumatoid factor (RF), assessed by indirect immunofluorescence and nephelometry, respectively, as part of standard rheumatologic work-up. Antiphospholipid antibody (aPL) status was recorded as a qualitative variable (positive/negative) according to routine institutional laboratory reports. Screening was performed using standard immunoassays employed in daily clinical practice, without subclass differentiation. The thrombophilia panel, when clinically indicated, included both genetic and biochemical parameters and was retrieved retrospectively for analysis. Genetic markers comprised Factor V Leiden (G1691A), Prothrombin (G20210A), MTHFR C677T, MTHFR A1298C, and Factor XIII (V34L) polymorphisms, identified using PCR-based genotyping methods. Measurements of Protein C, Protein S, and Antithrombin III were not available during the study period due to institutional test limitations and were therefore not included. Biochemical thrombophilia markers included plasma homocysteine, measured by enzymatic immunoassay, and lipoprotein(a), quantified using an immunoturbidimetric method. All laboratory analyses were conducted in a single institutional laboratory under standardized conditions with established internal and external quality controls. Reference intervals at our laboratory were as follows: ESR 0–22 mm/h, lipoprotein(a) 0–0.3 g/L, and plasma homocysteine 6–15 µmol/L. No additional blood sampling or laboratory testing was performed specifically for the purposes of this study.

### 2.6. Imaging Protocol

DSA was performed using transfemoral or radial-brachial access. Cerebral angiograms included aortography and selective catheterization of the common carotid, internal carotid, external carotid, subclavian, and vertebral arteries. Lesions were identified as elongated, smooth, non-calcified stenoses or occlusions, accompanied by post-stenotic dilatation, suggestive of inflammatory vasculitis. Collateral circulation patterns and hemodynamic flow characteristics were documented in every instance.

### 2.7. Statistical Examination

Data were analyzed utilizing SPSS version 22.0 (IBM Corp., Armonk, NY, USA). Continuous data were expressed as mean ± standard deviation (SD), whereas categorical variables were presented as number (%). The Kolmogorov–Smirnov test was employed to evaluate the normality of the distribution. Comparisons between groups were conducted using one-way ANOVA or the Kruskal–Wallis test for continuous variables, and Chi-square or Fisher’s exact test for categorical variables. A *p*-value less than 0.05 was deemed statistically significant. Due to the low prevalence of Takayasu arteritis, a formal a priori sample size calculation was not feasible at study initiation. All eligible patients with angiographically confirmed supra-aortic involvement presenting to our tertiary referral stroke center over the 8-year study period were consecutively included to minimize selection bias. Given the available cohort (21 stroke-positive and 25 stroke-negative TAK patients), post hoc assessment indicated that the study had limited statistical power (<50%) to detect small-to-moderate differences in the frequency of thrombophilia markers across groups. Therefore, the results should be interpreted as exploratory and hypothesis-generating rather than confirmatory.

### 2.8. Ethics Approval

This study was approved by the Selçuk University Local Ethics Committee (approval date: 4 November 2025; protocol number: 2025/631; official correspondence number: E-70632468-050.01-1122444). All procedures were conducted in accordance with the Declaration of Helsinki. Written informed consent was waived by the Ethics Committee due to the retrospective design of the study and the use of anonymized clinical data.

## 3. Results

### 3.1. Baseline Demographic and Clinical Characteristics

A total of 124 individuals were analyzed: 46 patients with angiographically confirmed Takayasu arteritis (21 with ischemic stroke and 25 without) and 78 healthy controls matched for age and sex. The average age did not exhibit significant variation among the groups (38.5 ± 7.7, 35.6 ± 8.6, and 36.2 ± 8.1 years, respectively; *p* = 0.66). Females constituted the majority in all cohorts (76.2%, 84.0%, and 71.8%; *p* = 0.48). No statistically significant variations were observed in traditional vascular risk variables, including hypertension, diabetes mellitus, dyslipidemia, atrial fibrillation, and smoking, across the three groups (all *p* > 0.05). Hypertension was more prevalent among controls (39.7%) compared to Takayasu patients (14.3% and 24.0%), indicating a nonsignificant trend (*p* = 0.09). Smoking was more common among stroke-positive Takayasu patients (57.1%) than among those without stroke (32.0%) or controls (47.4%), although this difference was not statistically significant (*p* = 0.19). The results suggest that cerebrovascular incidents in Takayasu arteritis are not predominantly attributed to traditional atherosclerotic risk factors (Table 1).

### 3.2. Inflammatory and Thrombophilia Markers, Angiographic Distribution, and Treatment Outcomes

Inflammatory activity, measured by ESR, was significantly higher in stroke-positive Takayasu patients (50.6 ± 11.4 mm/h) compared to stroke-negative cases (26.3 ± 10.6 mm/h) and controls (22.4 ± 8.4 mm/h), indicating a highly significant difference (*p* < 0.001). In the stroke-positive Takayasu subgroup, serum lipoprotein(a) levels were considerably elevated (23.7 ± 8.0 mg/dL) compared to stroke-negative patients (19.2 ± 4.2 mg/dL) and controls (18.3 ± 4.2 mg/dL) (*p* < 0.001). Lipoprotein(a) levels were numerically higher in stroke-positive patients (mean 23.7 mg/dL); however, all group means remained within the normal reference range (<30 mg/dL), and the clinical relevance of this difference is uncertain. Autoimmune markers were rarely positive and shown no significant change across groups: ANA positivity was 19.0%, 8.0%, and 9.0%; RF positivity was 9.5%, 4.0%, and 3.9%; and aPL positivity was 9.5%, 4.0%, and 3.8%, respectively (all *p* > 0.05). Classical genetic thrombophilia variations were uniformly distributed throughout the groups. The prevalence rates of Factor V Leiden (14.3%, 4.0%, 11.5%), Prothrombin G20210A (4.8%, 12.0%, 10.3%), MTHFR C677T (33.3%, 20.0%, 24.4%), MTHFR A1298C (9.5%, 12.0%, 9.0%), and Factor XIII V34L (23.8%, 16.0%, 16.7%) exhibited no significant differences (all *p* > 0.05). The comparison of plasma homocysteine positivity among groups was assessed using the Chi-square test (χ^2^ = 0.18, *p* = 0.91), revealing no statistically significant difference. These data indicate that systemic inflammation and elevated lipoprotein(a), rather than hereditary thrombophilia, are the primary laboratory characteristics linked to cerebrovascular manifestations in Takayasu arteritis (Table 2).

**Figure 2:**
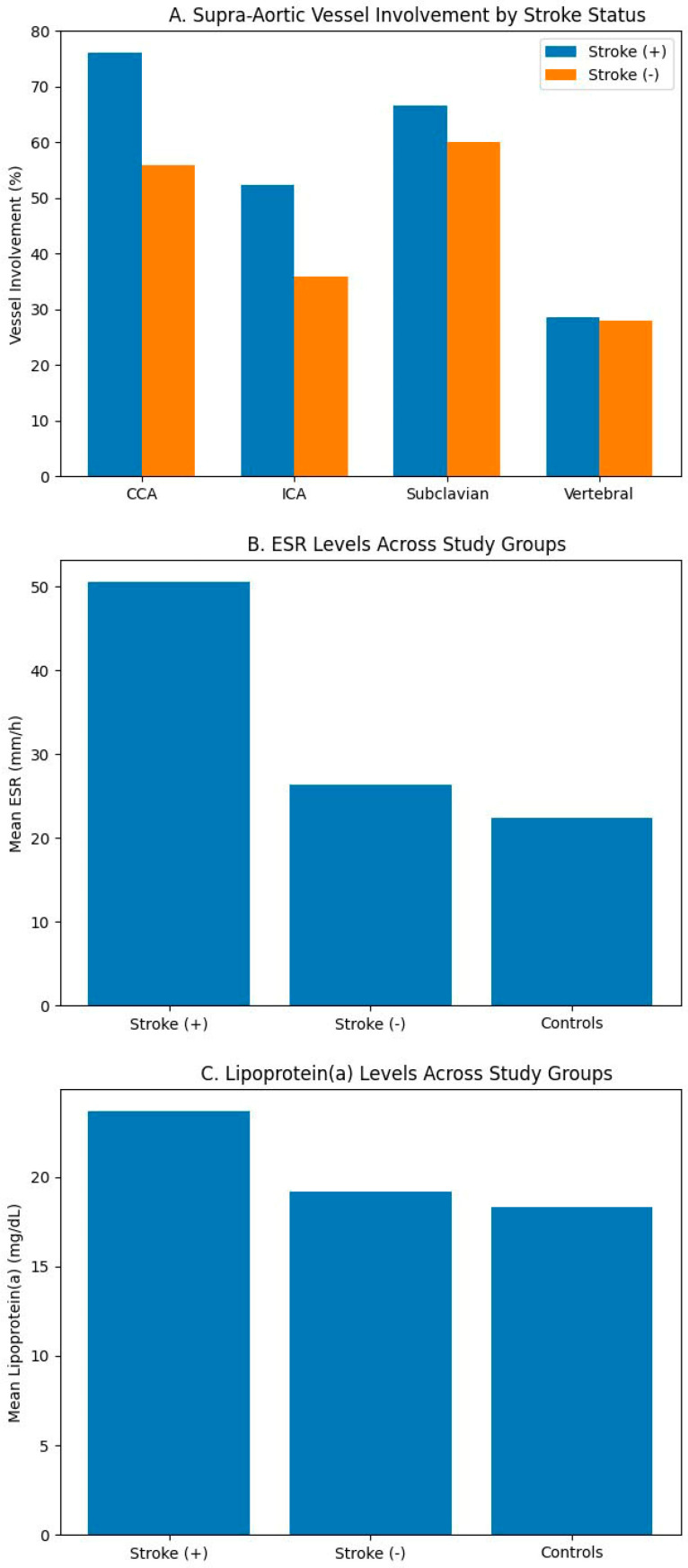
Angiographic and Laboratory Comparisons According to Stroke Status in Supra-Aortic Takayasu Arteritis. (**A**) Distribution of supra-aortic arterial involvement stratified by stroke status. The figure illustrates the frequency of common carotid artery (CCA), internal carotid artery (ICA), subclavian artery, and vertebral artery involvement in stroke-positive and stroke-negative Takayasu arteritis patients. Stroke-positive patients demonstrate a numerically higher burden of occlusive disease, particularly within the CCA and ICA territories. (**B**) Comparison of erythrocyte sedimentation rate (ESR) levels across stroke-positive Takayasu arteritis, stroke-negative Takayasu arteritis, and control groups. Markedly elevated inflammatory activity is observed in the stroke-positive subgroup. (**C**) Comparison of lipoprotein(a) levels across the same three groups. Although statistical differences were observed, mean values in all groups remained within laboratory reference ranges, suggesting limited clinical elevation.

Extensive supra-aortic arterial involvement was observed in both groups, with a higher burden of occlusive lesions among stroke-positive patients. In the stroke group: Common carotid artery (CCA) involvement was present in 76.2% (3 stenosis, 13 occlusion). Internal carotid artery (ICA) lesions were seen in 52.4% (2 stenosis, 9 occlusion). Subclavian artery disease occurred in 66.7% (7 stenosis, 7 occlusion). Vertebral artery involvement was present in 28.6% (2 stenosis, 4 occlusion). Intracranial disease was less frequent, including right ACA occlusion (n = 1) and MCA involvement (left MCA stenosis n = 2; right MCA stenosis/occlusion n = 3). Stroke-negative patients showed a similar but slightly less extensive distribution of disease, with CCA (56%), ICA (36%), subclavian (60%), and vertebral (28%) involvement (Table 3). Overall, both groups demonstrated multi-territorial supra-aortic vasculopathy consistent with hemodynamic compromise patterns (Figure 2).

Treatment characteristics and functional outcomes are summarized in Table 4. Among the 46 patients with supra-aortic Takayasu arteritis, treatment strategies differed according to stroke status and timing of presentation. All patients (46/46, 100%) received systemic corticosteroid therapy, initiated at approximately 1 mg/kg/day of oral prednisone equivalent following acute-phase management when applicable. Of the 21 stroke-positive patients, 4 (19.0%) presented during the acute phase. Among these, 2 patients received intravenous thrombolysis in accordance with standard stroke protocols, whereas 2 were not eligible. All acute stroke patients received dual antiplatelet loading and high-dose intravenous pulse corticosteroid therapy, followed by transition to oral corticosteroids. During the subacute period, conventional steroid-sparing immunosuppressive therapy (methotrexate, azathioprine, or mycophenolate mofetil) was initiated in 2 of the acute stroke patients, while 2 patients with more severe neurological involvement required escalation to biologic therapy with the interleukin-6 receptor inhibitor tocilizumab. The remaining 17 stroke-positive patients who presented outside the acute phase were either already receiving or were newly started on oral corticosteroids combined with conventional immunosuppressive agents. Overall, 19 of 21 stroke-positive patients (90.5%) received conventional immunosuppressive therapy, and 2 patients (9.5%) required biologic escalation. Among the 25 stroke-negative patients, 3 were receiving long-term immunosuppressive therapy at presentation, while 22 were newly initiated on corticosteroids in combination with a conventional immunosuppressive agent, resulting in 22 of 25 patients (88.0%) receiving steroid-sparing immunosuppression. No stroke-negative patients required biologic therapy. Delayed endovascular intervention (balloon angioplasty or stenting) was performed in 3 of the 4 acute stroke patients and 12 of the 17 non-acute stroke patients, yielding a total of 15 stroke-positive individuals (71.4%) undergoing revascularization. In the stroke-negative group, 3 patients with critical supra-aortic stenosis required delayed endovascular treatment. No endovascular procedures were performed during the acute inflammatory phase. All patients who underwent stenting received dual antiplatelet therapy (aspirin 100 mg/day and clopidogrel 75 mg/day) for a minimum of three months, followed by transition to long-term single antiplatelet therapy, which was recommended for all patients irrespective of stroke status.

## 4. Discussion

Takayasu arteritis predominantly affects young women and represents an important non-atherosclerotic cause of ischemic stroke in this population [4]. In patients with supra-aortic involvement, inflammatory large-vessel stenosis leads to impaired cerebral inflow and hemodynamic compromise, occasionally accompanied by thromboembolic phenomena [6,7]. Although hereditary and acquired thrombophilia are recognized contributors to stroke in young adults [15], their additive role in the inflammatory and flow-compromised setting of Takayasu arteritis remains insufficiently defined [10]. The present study therefore evaluated whether genetic or acquired prothrombotic factors independently contribute to ischemic risk in angiographically confirmed supra-aortic Takayasu arteritis. Importantly, the principal objective of this study was to explore differences between stroke-positive and stroke-negative Takayasu patients. Comparisons with controls were intended to contextualize laboratory distributions rather than to imply that Takayasu arteritis per se alters thrombophilia prevalence.

Our findings are consistent with the established epidemiological profile of Takayasu arteritis, characterized by early adult onset and female predominance [3,16]. Traditional vascular risk factors were relatively infrequent in our cohort, supporting the concept that cerebrovascular events in this population are more closely linked to inflammatory large-vessel remodeling and hemodynamic compromise than to classical atherosclerotic mechanisms. However, these observations remain descriptive and do not imply causality.

All patients demonstrated non-atherosclerotic supra-aortic stenosis or occlusion confirmed by DSA. ESR levels were significantly elevated in the Takayasu cohort compared to controls and were highest among stroke-positive patients, suggesting that active systemic inflammation may contribute to hemodynamic deterioration and ischemic vulnerability. This observation aligns with prior studies showing that elevated acute-phase reactants are associated with progression of arterial lesions and increased risk of cerebrovascular events in Takayasu arteritis [6,17].

These findings support the concept that active inflammation, rather than fixed fibrotic damage, is the predominant driver of hemodynamic impairment in Takayasu arteritis [5]. Inflammatory vascular remodeling disrupts laminar flow and promotes endothelial dysfunction, thereby increasing ischemic susceptibility [18]. Clinically, this underscores the importance of close monitoring of inflammatory activity using ESR and CRP, complemented by imaging modalities such as PET or MRI angiography, to guide timely immunosuppressive therapy and risk stratification [19].

In accordance with previous research, rheumatologic autoantibodies, including ANA and RF, were predominantly negative or detected at low titers in our cohort, reinforcing the notion that Takayasu arteritis is a non-autoantibody mediated vasculitis primarily influenced by cellular immune mechanisms rather than humoral autoimmunity. Prior studies by Aydin et al. [20] and Maffei et al. [21] similarly reported ANA positivity rates under 20% and RF positivity rates below 10%, suggesting that these markers possess neither diagnostic nor pathogenic relevance in Takayasu arteritis.

A key aspect of our study was the systematic evaluation of hereditary thrombophilia markers in angiographically confirmed supra-aortic Takayasu arteritis. The prevalence of Factor V Leiden, Prothrombin G20210A, MTHFR polymorphisms, Factor XIII (V34L), and elevated homocysteine levels did not differ significantly between patients and controls, consistent with prior reports in large-vessel vasculitis [12]. Although carrier rates of Factor V Leiden and Prothrombin G20210A appeared relatively high in both groups, these frequencies are comparable to those reported in Mediterranean populations and likely reflect regional genetic background rather than disease-specific enrichment [22].

Elevated homocysteine levels (>15 µmol/L) were common in both Takayasu arteritis and control groups. All measurements were obtained after standardized fasting and analyzed under uniform laboratory conditions, minimizing pre-analytical variability. The high prevalence likely reflects regional dietary and genetic influences reported in Mediterranean populations [22]. Importantly, homocysteine levels were not associated with ischemic presentation in our cohort, suggesting that this finding represents a background metabolic pattern rather than a disease-specific cerebrovascular risk factor in Takayasu arteritis.

Although hypercoagulability has been proposed as a contributor to vascular occlusion in Takayasu arteritis, the evidence remains inconsistent. Elevated homocysteine and PAI-1 levels observed during active disease likely reflect secondary inflammatory effects rather than primary hereditary thrombophilia [23]. Prior studies similarly suggest that thrombosis in vasculitis is more closely linked to cytokine-mediated endothelial injury and flow disturbance than to genetic mutations [11]. In line with these observations, the higher ESR levels in our stroke-positive cohort indicate that active inflammation and hemodynamic compromise, rather than inherited thrombophilia, are the principal drivers of cerebrovascular ischemia in this population. Clinically, these findings support prioritizing inflammation control and vascular monitoring, with selective thrombophilia screening reserved for atypical or recurrent thrombotic presentations.

Our findings further indicate that classical inherited thrombophilia markers including Factor V Leiden, Prothrombin G20210A, MTHFR polymorphisms, and Factor XIII V34L are not significantly associated with vascular events in Takayasu arteritis, consistent with prior reports in large-vessel vasculitis [10,11,12]. The inclusion of antiphospholipid antibodies and lipoprotein(a) addressed gaps in earlier studies; however, aPL positivity was rare and lipoprotein(a) levels, although numerically higher in stroke-presenting patients, remained within the normal range, suggesting no clinically meaningful prothrombotic effect. Overall, these data support the concept that thrombotic complications in Takayasu arteritis are more closely related to inflammation-driven endothelial activation and hemodynamic stress than to inherited prothrombotic predisposition. But, given the number of laboratory and genetic variables examined, the risk of inflated type I error due to multiple comparisons must be considered. Although nominal *p*-values were reported, the analyses were exploratory in nature. After correction using a false discovery rate approach, the association of ESR remained robust, whereas other findings, including Lp(a), should be interpreted cautiously as hypothesis-generating. These results warrant validation in larger cohorts.

Given the rarity of Takayasu arteritis and the limited size of genetically stratified subgroups, several between group comparisons did not reach statistical significance. Nevertheless, the consistent pattern across all evaluated markers showing similar or lower thrombophilia rates in Takayasu patients compared with controls supports the internal coherence of our findings. The use of age- and sex-matched controls and standardized laboratory analyses strengthens the validity of these comparisons despite the modest sample size. Furthermore, prior studies from Türkiye and East Asia have demonstrated that arch-branch involvement (Type I) carries the highest association with cerebrovascular ischemia, with reported stroke/TIA rates of approximately 25–30% in such cohorts [5,24]. The stroke-focused referral profile of our center may have contributed to the relatively high incidence observed in our sample.

Angiographic analysis revealed a higher frequency of multi-territorial occlusive lesions in stroke positive patients, particularly involving the common and internal carotid arteries and subclavian arteries, whereas stenotic lesions were similarly distributed across groups. Although causal inference cannot be established, the predominance of proximal inflow occlusions suggests that structural hemodynamic compromise may be a key determinant of cerebrovascular vulnerability in supra-aortic Takayasu arteritis. Intracranial involvement was uncommon and did not account for most ischemic events, supporting a mechanism primarily related to large vessel inflow limitation.

In the comparison between stroke and non-stroke subgroups, age, sex, and traditional vascular risk factors were similar, whereas ESR was significantly higher in stroke positive patients. This finding aligns with prior reports linking elevated inflammatory markers to progression of luminal lesions and increased ischemic risk in Takayasu arteritis [5]. Mechanistically, heightened inflammatory activity may exacerbate endothelial dysfunction and reduce cerebrovascular reserve, thereby lowering the threshold for hemodynamic stroke in the setting of critical supra-aortic flow limitation.

Treatment patterns in this supra-aortic Takayasu cohort reflected the central role of inflammation and hemodynamic compromise in cerebrovascular events. Stroke positive patients required more intensive therapy, including dual antiplatelet loading and high-dose corticosteroids in the acute phase. Standard reperfusion strategies, including intravenous thrombolysis, were selectively applied when imaging excluded high-risk vascular pathology. Immunosuppressive therapy was broadly implemented across groups, underscoring the importance of sustained disease control, while biologic agents were reserved for severe or refractory cases. Endovascular interventions were performed only after inflammatory stabilization, with higher rates among stroke-positive patients reflecting greater structural flow limitation. Although hereditary thrombophilia was not prominent, long-term antiplatelet therapy remained integral to secondary prevention. Overall, these findings highlight that stroke prevention in Takayasu arteritis primarily relies on inflammation control and appropriately timed revascularization.

Clinical outcomes in our cohort were favorable, with no mortality and functional independence (mRS 0–2) achieved in 81% of stroke-positive patients. These results compare favorably with previously reported Takayasu stroke series, which have described higher disability rates [2,5]. The combination of early high-dose corticosteroid therapy, timely initiation of immunosuppression, and delayed endovascular intervention after inflammatory stabilization may have contributed to these outcomes. Consistent with prior recommendations, revascularization was performed only after disease activity was controlled, minimizing procedural risk. Overall, these findings underscore the importance of inflammation control and carefully timed intervention in optimizing neurological outcomes in supra-aortic Takayasu arteritis.

Smoking was more frequent among stroke-positive patients, although the difference did not reach statistical significance. Prior studies in vasculitis suggest that smoking may exacerbate endothelial dysfunction and inflammatory vascular injury [25]. While our data do not establish a causal relationship, this trend supports the potential contribution of modifiable environmental factors in patients with flow-limiting supra-aortic disease. Regarding the control group, although individuals were selected to be free of systemic inflammatory or thrombotic disorders, the relatively high prevalence of hypertension (39.7%) suggests that they may not represent an ideal community-based healthy cohort. This likely reflects hospital-based recruitment and introduces potential selection bias. Nevertheless, the absence of supra-aortic vascular abnormalities or prior ischemic events preserved their suitability for comparative thrombophilia analyses. We acknowledge that the elevated hypertension rate and lack of vascular imaging in controls limit generalizability and should be considered when interpreting intergroup comparisons.

Our findings indicate that in supra-aortic Takayasu arteritis, systemic inflammation and hemodynamic compromise are the primary determinants of cerebrovascular events, whereas hereditary thrombophilia appears to play a limited role. These results support prioritizing aggressive control of vascular inflammation and close monitoring of arterial patency and cerebral perfusion as central components of stroke prevention. Routine thrombophilia screening may not be necessary for all patients but can be considered selectively in cases of recurrent thrombosis, ischemia during apparent remission, or suggestive family history. Overall, a management strategy focused on immunomodulation and timely vascular surveillance is more justified than extensive genetic testing in this population.

### Strengths and Limitations

This study has several strengths, including the exclusive enrollment of patients with angiographically verified supra-aortic Takayasu arteritis, the use of age- and sex-matched healthy controls, and the comprehensive evaluation of genetic, immunologic, and biochemical thrombophilia markers under standardized laboratory conditions. The uniform fasting protocol and centralized analytic methodology further strengthen the internal validity of the laboratory comparisons.

However, several limitations must be acknowledged. This was a retrospective, single-center study with a relatively small sample size due to the rarity of supra-aortic Takayasu arteritis, which limits both statistical power and external generalizability. The study may therefore be underpowered to detect small-to-moderate differences in low-frequency genetic polymorphisms or uncommon prothrombotic markers, raising the possibility of type II error. Accordingly, the thrombophilia findings should be interpreted as exploratory and hypothesis-generating rather than definitive. The absence of vascular imaging in the control group limits certainty regarding subclinical cerebrovascular pathology. Additionally, the hospital-based control group demonstrated a relatively high prevalence of hypertension for its age, suggesting a cardiovascular-enriched reference population rather than a community-derived healthy sample. This may have increased the baseline prevalence of vascular or prothrombotic markers and potentially attenuated between-group differences, biasing results toward the null hypothesis. Functional coagulation assays were not performed, and the thrombophilia panel did not include Protein C, Protein S, or Antithrombin III levels, limiting full characterization of inherited prothrombotic states. Furthermore, reliable disease-duration data were unavailable in a subset of patients diagnosed at first presentation to our center. Therefore, non-significant findings, particularly within the thrombophilia panel, should be interpreted cautiously. Larger, multicenter studies with population-based controls are warranted to clarify the potential contribution of subtle thrombophilic factors to cerebrovascular risk in Takayasu arteritis. An additional limitation is the reliance on ESR as the primary inflammatory marker. ESR is a non-specific and time-lagged acute-phase reactant influenced by demographic and hematologic variables, particularly relevant in young women with autoimmune disease. CRP, a more specific and rapidly responsive biomarker, was not consistently available in this retrospective cohort, and IL-6 was not routinely measured. Therefore, the assessment of inflammatory activity should be interpreted cautiously, and prospective studies with standardized CRP and cytokine profiling are needed.

## 5. Conclusions

Overall, our findings suggest that inflammation-related vascular changes and hemodynamic impairment are more closely associated with stroke in supra-aortic Takayasu arteritis than hereditary thrombophilia. However, given the cross-sectional design, these relationships should be interpreted as associations rather than causal mechanisms. Thrombophilia screening may be reserved for patients with recurrent or unexplained ischemia, while clinical management should prioritize timely vascular evaluation, strict inflammation control, and modification of risk factors such as smoking.

## Figures and Tables

**Table 1:** Demographic and Clinical Characteristics of the Study Groups.

Variable	Stroke (+) Takayasu (n = 21)	Stroke (−) Takayasu (n = 25)	Control (n = 78)	*p*-Value	Interpretation
Age (years)	38.52 ± 7.73	35.56 ± 8.65	36.17 ± 8.06	0.66	No significant difference
Female sex (%)	16 (76.2)	21 (84.0)	56 (71.8)	0.48	NS
Hypertension (%)	3 (14.3)	6 (24.0)	31 (39.7)	0.09	Trend toward higher prevalence in controls
Diabetes mellitus (%)	1 (4.8)	5 (20.0)	10 (12.8)	0.29	NS
Dyslipidemia (%)	4 (19.0)	4 (16.0)	15 (19.2)	0.96	NS
Smoking (%)	12 (57.1)	8 (32.0)	37 (47.4)	0.19	Slightly more frequent in stroke (+) group
Atrial fibrillation (%)	0 (0)	1 (4.0)	6 (7.7)	0.37	NS

Comparison of demographic and clinical parameters among patients with stroke-positive Takayasu arteritis, stroke-negative Takayasu arteritis, and healthy controls. Data are expressed as mean ± standard deviation for continuous variables and as n (%) for categorical variables. Between-group differences were assessed using one-way ANOVA for continuous variables and the Chi-square or Fisher’s exact test for categorical variables. A *p*-value < 0.05 was considered statistically significant. NS: Not Significant.

**Table 2:** Laboratory and Genetic Parameters among Groups.

Variable	Stroke (+) Takayasu (n = 21)	Stroke (−) Takayasu (n = 25)	Control (n = 78)	*p*-Value	Interpretation
Erythrocyte Sedimentation Rate (mm/h)	50.62 ± 11.42	26.32 ± 10.60	22.38 ± 8.43	<0.001	Significantly higher in Stroke (+) Takayasu group
Lipoprotein (a) (mg/dL)	23.68 ± 8.01	19.23 ± 4.16	18.27 ± 4.19	<0.001	Significantly higher in Stroke (+) Takayasu group
ANA positive (%)	4 (19.0)	2 (8.0)	7 (9.0)	0.42	NS
RF positive (%)	2 (9.5)	1 (4.0)	3 (3.9)	0.65	NS
aPL positive (%)	2 (9.5)	1 (4.0)	3 (3.8)	0.56	NS
Factor V Leiden (%)	3 (14.3)	1 (4.0)	9 (11.5)	0.52	NS
Prothrombin G20210A (%)	1 (4.8)	3 (12.0)	8 (10.3)	0.74	NS
MTHFR C677T (%)	7 (33.3)	5 (20.0)	19 (24.4)	0.61	NS
MTHFR A1298C (%)	2 (9.5)	3 (12.0)	7 (9.0)	0.94	NS
Factor XIII (V34L) (%)	5 (23.8)	4 (16.0)	13 (16.7)	0.75	NS
Homocysteine elevated (%)	5 (23.8)	7 (28.0)	22 (28.2)	0.91	NS

Comparison of laboratory inflammatory markers and genetic thrombophilia profiles among stroke-positive Takayasu arteritis, stroke-negative Takayasu arteritis, and control groups. Data are presented as mean ± standard deviation or as n (%). One-way ANOVA was used for continuous variables, and Chi-square or Fisher’s exact tests were applied for categorical data. ESR and lipoprotein(a) levels were significantly elevated in the stroke-positive Takayasu subgroup, whereas the distribution of classical thrombophilia mutations and autoantibody markers (ANA, RF, aPL) did not differ significantly among groups. Statistical significance was defined as *p* < 0.05. NS: Not Significant.

**Table 3:** Angiographic Features of 46 Patients with Supra-Aortic Takayasu Arteritis.

Vessel Involvement	Stroke (+) n = 21	%	Stroke (−) n = 25	%
Common Carotid Artery (CCA)	16	76.2%	14	56.0%
Stenosis	3	14.3%	3	12.0%
Occlusion	13	61.9%	11	44.0%
Internal Carotid Artery (ICA)	11	52.4%	9	36.0%
Stenosis	2	9.5%	2	8.0%
Occlusion	9	42.9%	7	28.0%
Subclavian Artery	14	66.7%	15	60.0%
Stenosis	7	33.3%	7	28.0%
Occlusion	7	33.3%	8	32.0%
Vertebral Artery	6	28.6%	7	28.0%
Stenosis	2	9.5%	4	16.0%
Occlusion	4	19.0%	3	12.0%
ACA (Right)	1	4.8%	0	0%
ACA (Left)	0	0%	0	0%
MCA (Left)	2	9.5%	1	4.0%
MCA (Right)	3	14.3%	0	0%

The table summarizes vessel-specific involvement patterns in patients with stroke-positive (n = 21) and stroke-negative (n = 25) Takayasu arteritis. For each arterial segment, both the frequency and distribution of stenotic and occlusive lesions are presented. Data reflect digital subtraction angiography (DSA) findings obtained at diagnosis or first vascular evaluation. Percentages are calculated within each clinical subgroup. ACA = anterior cerebral artery; MCA = middle cerebral artery.

**Table 4:** Treatment Profile and Functional Outcomes in Supra-Aortic Takayasu Arteritis.

Treatment Parameter	Stroke (+) (n = 21)	Stroke (−) (n = 25)	Total (N = 46)
Acute stroke presentation	4 (19.0%)	–	4 (8.7%)
-Received IV thrombolysis	2 (9.5%)	–	2 (4.3%)
-No thrombolysis	2 (9.5%)	–	2 (4.3%)
Antiplatelet loading (ASA 300 mg + CLOP 450 mg)	4 (19.0%)	–	4 (8.7%)
Pulse steroid (5 days IV)	4 (19.0%)	–	4 (8.7%)
Maintenance steroid (1 mg/kg/day)	21 (100%)	22 (88.0%)	43 (93.5%)
Immunosuppressive therapy (any)	21 (100%)	25 (100%)	46 (100%)
-MTX/AZA/MMF	19 (90.5%)	22 (88.0%)	41 (89.1%)
-Tocilizumab	2 (9.5%)	0 (0%)	2 (4.3%)
Endovascular treatment (balloon/stent)	15 (71.4%)	3 (12.0%)	18 (39.1%)
-Acute-phase stenting	0 (0%)	0 (0%)	0 (0%)
-Delayed stenting ≥1 month	15 (71.4%)	3 (12.0%)	18 (39.1%)
DAPT after stenting (ASA 100 mg + CLOP 75 mg ≥ 3 months)	15 (71.4%)	3 (12.0%)	18 (39.1%)
Long-term single antiplatelet	21 (100%)	25 (100%)	46 (100%)
Functional outcome (mRS 0–2)	17 (81.0%)	–	17 (37.0%)
Functional outcome (mRS 3–5)	4 (19.0%)	–	4 (8.7%)

The table summarizes acute-phase management, immunosuppressive therapy, antiplatelet regimens, and endovascular interventions among stroke-positive (n = 21) and stroke-negative (n = 25) patients. Acute ischemic stroke cases received standardized dual antiplatelet loading and pulse corticosteroids, whereas endovascular procedures were performed only after clinical stabilization and reduction in inflammatory activity. The distribution of delayed stenting, dual antiplatelet therapy, and long-term single antiplatelet use is presented for both groups. Functional outcomes in the stroke subgroup are shown using the modified Rankin Scale (mRS), with the proportion achieving functional independence (mRS 0–2) and those with residual disability (mRS 3–5). MTX: Methotrexate, AZA: Azathioprine, MMF: Mycophenolate mofetil.

## Data Availability

The datasets generated and analyzed during the current study are available from the corresponding author on reasonable request.
